# An interpretable machine learning screening model for MoCA-defined possible mild cognitive impairment in rural Xinjiang: a preliminary framework for resource-limited primary care

**DOI:** 10.3389/fpubh.2026.1854956

**Published:** 2026-08-26

**Authors:** Yutong Li, Lili He, Jiahuan He, Ting Liang, Chun Ji, Zhiyan Zhang, Jieting Chen, Pengxiang Zuo

**Affiliations:** 1 School of Medicine, Shihezi University, Shihezi, China; 2 Hospital of the 51st Regiment, Tumushuke, China

**Keywords:** machine learning, mild cognitive impairment, primary care, rural Xinjiang, screening model

## Abstract

**Objective:**

To construct and validate an interpretable machine learning screening model for MoCA-defined possible mild cognitive impairment applicable to residents in rural areas of Xinjiang, China.

**Methods:**

A total of 708 residents from rural Xinjiang were recruited between June and July 2025. Six machine learning methods—Logistic Regression (LR), Adaptive Boosting (AdaBoost), Multilayer Perceptron (MLP), Naïve Bayes (NB), Extreme Gradient Boosting (XGBoost), and Light Gradient Boosting Machine (LightGBM)—were employed to identify MoCA-defined possible MCI based on data from four dimensions: physiological, psychological, social, and behavioral. The optimal model was selected using the Area Under the Curve (AUC) as the primary evaluation metric. Model interpretability was assessed using SHapley Additive explanations (SHAP), and the dose–response relationships between continuous variables and MoCA-defined possible MCI were visualized using Restricted Cubic Splines (RCS).

**Results:**

After feature selection, nine key variables were retained for model construction. Among the six models developed, the XGBoost model demonstrated the best performance, achieving an AUC of 0.839 (95% CI: 0.811–0.867) in the training set and 0.747 (95% CI: 0.673–0.820) in the validation set. SHAP analysis revealed that Education, Age, and Direct Bilirubin (DBIL) were the three most influential predictors. Restricted cubic spline analysis indicated linear correlations with MoCA-defined possible MCI for Education, Age, DBIL, and Systolic Blood Pressure (SBP) (overall *p* < 0.05, non-linear *p* > 0.05). A non-linear association was observed for Triglycerides (TG) (overall *p* < 0.001, non-linear *p* < 0.001), with its dose–response curve exhibiting a complex non-linear trend.

**Conclusion:**

We developed and validated an interpretable screening model for MoCA-defined possible MCI tailored to rural Xinjiang by integrating established machine learning techniques within a localized framework. The primary contribution of this work lies in the applied and translational validation of these methods for an understudied, resource-limited population rather than in algorithmic innovation. The finalized XGBoost model requires only nine easily obtainable variables, offering moderate discriminative performance and useful interpretability for preliminary screening purposes. This preliminary screening framework may offer a potentially useful approach for cognitive risk stratification in resource-limited primary care settings, though independent external validation is required before broader implementation.

## Introduction

Mild cognitive impairment (MCI) represents an intermediate transitional state between normal aging and dementia, characterized primarily by a mild but measurable and persistent decline in cognitive domains such as memory, executive function, language, and visuospatial skills (1). It is recognized as an early stage in the Alzheimer’s disease continuum, and early identification is crucial for timely intervention and delaying disease progression (2). The core clinical features involve cognitive decline exceeding that of normal aging yet not meeting the diagnostic criteria for dementia, with relatively preserved activities of daily living, often accompanied by mild neuropsychiatric symptoms (3). The risk of progression to dementia is significantly elevated, leading to decreased quality of life and increased socio-economic and healthcare burdens (4). Economic evaluations from the German DELCODE (5)cohort study and the US-based GERAS-US (6) prospective study consistently demonstrate that MCI imposes significant societal costs, and both direct non-medical costs and caregiver burden escalate sharply with progression to dementia, confirming a clear positive correlation between disease stage and economic burden. This growing economic pressure is not confined to the individual level but reflects the high global prevalence of MCI. An epidemiological meta-analysis indicated an overall prevalence of 19.7% in individuals over 50, rising to 32.1% after 2019, with regional estimates around 19.8% in Asia and 23.1% in Africa (7). Facing this widespread public health challenge, the onset and progression of MCI are not attributable to a single etiology but adhere to a biopsychosocial multidimensional model, with 14 modifiable risk factors identified, the elimination of which could potentially prevent or delay up to 45% of dementia cases (8).

Given this disease burden and potential for intervention, with the ongoing deepening of global population aging, early identification of high-risk groups for MCI and the formulation of targeted prevention and control strategies to delay or prevent progression to dementia have become a pressing medical and public health issue (9). Recent advances in neuroscience have progressively elucidated typical pathological mechanisms of common cognitive disorders, including abnormal amyloid-beta (Aβ) deposition in cerebrospinal fluid (10), elevated phosphorylated Tau protein (11), and genetic susceptibility linked to the APOEε4 allele (12). Emerging evidence also indicates that neuroimaging markers, such as hippocampal volume atrophy on structural MR (13) and Aβ deposition visualized by amyloid PET imaging (14), provide objective evidence of brain neurodegeneration. However, detecting such biomarkers is often costly and invasive, limiting their applicability in primary care settings. More importantly, they primarily reflect established neuropathological changes that are typically difficult to reverse through conventional interventions. This limitation has shifted research focus toward more plastic and modifiable risk factors. Beyond immutable factors like age (15) and sex (16), ample evidence demonstrates that physiological conditions such as hypertension (17) and diabetes (18), as well as psychological states like anxiety (19) and depression (20), significantly impact cognitive health. Furthermore, some studies indicate that adverse behavioral habits such as high-sugar diets (21) and smoking (22), along with social factors like marital status (23) and low income (24), also significantly increase the risk of MCI. These findings align closely with the comprehensive intervention strategies advocated in the World Health Organization (WHO) Guidelines on Risk Reduction of Cognitive Decline and Dementia (25). These guidelines emphasize multi-domain, integrated measures for early screening and personalized interventions in high-risk populations. However, implementation faces significant challenges in low- and middle-income countries and rural areas due to limited healthcare resources—a resource constraint that has been a primary driver for our focus on developing a tool suitable for primary care.

Although research has begun to address MCI risk in Chinese populations, systematic exploration targeting rural areas, particularly resource-limited western regions, remains insufficient (26). Existing screening models are predominantly developed based on populations from Western high-income countries (27), and their applicability in rural western China is unclear. While some domestic models focus on rural populations, they often rely on complex biomarkers like cerebrospinal fluid analysis or cranial MRI (28), hindering their implementation in primary care settings. Moreover, relevant domestic research has largely concentrated on eastern regions (29), with less attention paid to the multi-ethnic, socioeconomically distinct, and unique chronic disease comorbidity patterns of rural Xinjiang (30), where early identification and risk assessment for MCI remain critical challenges. Commonly used screening tools in this region (31), while having broad applicability, are often unidimensional in their assessment (32) and may fail to capture the complex interplay of local risk factors, thereby limiting their precision and efficacy for early intervention. Consequently, a customized screening tool capable of integrating multidimensional risk factors and tailored to the local context is urgently needed in rural settings with constrained healthcare resources.

Therefore, this study, based on cross-sectional survey data from rural Xinjiang, China, employs LASSO regression for variable selection and utilizes machine learning algorithms to construct a screening model for MoCA-defined possible MCI. Furthermore, we incorporate explainable artificial intelligence techniques like SHAP to elucidate the influence mechanisms of various risk factors and explore potential non-linear relationships. The objective is to provide a simple, applicable, and clinically feasible early screening tool for MoCA-defined possible MCI in primary care settings in rural Xinjiang, thereby offering data support and practical evidence for regional cognitive health promotion and disease prevention.

## Methods

### Study design

This study employed a cross-sectional design based on residents of rural Xinjiang, China. The survey was conducted across five villages in the Xinjiang region between June and July 2025. Based on expert opinion and extensive literature review, data covering physiological, psychological, social, and behavioral dimensions were collected through face-to-face interviews and physical examinations. Six machine learning methods were applied to construct a screening model for MoCA-defined possible MCI for residents in rural Xinjiang. Model performance was compared to select the optimal model, which was subsequently interpreted using SHAP. Additionally, Restricted Cubic Splines (RCS) were employed to visualize the dose–response relationships between continuous variables and MoCA-defined possible MCI. The study protocol was approved by the Medical Ethics Committee of the First Affiliated Hospital of Shihezi University (Approval No.: KJ2025-332-02), and the research was conducted in strict adherence to the ethical principles of the Declaration of Helsinki. This study strictly followed the Transparent Reporting of a Multivariable Prediction Model for Individual Prognosis or Diagnosis–Artificial Intelligence (TRIPOD+AI) statement for the development, validation, and performance evaluation of the screening model (33).

### Participants

Using a cluster random sampling approach, five administrative villages in Tumxuk City were selected as survey sites. Recruitment was embedded within local annual universal health examination services. Researchers collaborated with village clinicians to screen all registered residents via clinic archives. After participants finished all routine physical tests on the examination day, trained investigators introduced the study and invited voluntary MoCA assessment. A total of 711 residents completed full physical examination records; 708 participants completed the complete cognitive evaluation with a response rate of 99.6%, while only three individuals declined participation. The dataset was divided into training and validation groups via stratified random sampling based on the binary outcome indicator. A CONSORT-style participant flow diagram is provided in supplementary Figure S1. Inclusion criteria were: (a) age ≥ 18 years; (b) permanent resident of the community (residing for ≥ 6 months); (c) voluntary participation and provision of written informed consent. Exclusion criteria included: (a) previously diagnosed dementia; (b) history of psychotropic drug dependence or substance abuse; (c) severe visual, auditory, or speech impairments hindering assessment cooperation; (d) voluntary withdrawal from the study during the interview process.

### Sample size

We adopted the sample size calculation framework proposed by Riley (34) for binary outcome prediction model development, which sets three core evaluation standards: shrinkage factor ≥ 0.9, minor discrepancy between apparent and adjusted R^2^, and risk estimation error ≤ 0.05. Assuming a conservative national prevalence of 20.8% (35) and a Cox-Snell R^2^ of 0.15 with 9 predictors, the theoretical minimum sample size required was 494 participants. Our training dataset contained 497 individuals with 368 cases of MoCA-defined possible MCI, which fully satisfied the statistical power threshold. To further reduce overfitting risks, LASSO feature screening, 10-fold cross-validation, XGBoost regularization and SMOTE resampling were all implemented exclusively within training folds (36).

### Outcome

MoCA-defined possible MCI was assessed using the Montreal Cognitive Assessment (MoCA) scale. This standardized cognitive screening tool encompasses seven cognitive domains: visuospatial/executive function, naming, attention, language, abstraction, delayed recall, and orientation. The total score ranges from 0 to 30 (37). According to domestic norms and cultural adaptations, one point was added to the raw score for participants with ≤ 12 years of education to correct for educational bias (38). An adjusted total score of ≤ 25 was classified as MoCA-defined possible MCI. The MoCA scale only acts as a preliminary screening instrument rather than a gold-standard clinical diagnostic criterion. In this study, the MoCA score was converted into a binary categorical variable serving as the outcome measure.

### Potential predictors

This study systematically collected 33 feature variables across four dimensions—physiological, psychological, behavioral, and social—using standardized questionnaires and physical measurements. Specifically, the physiological dimension included basic demographic information (Sex, Age), anthropometric indices (Waist Circumference, Hip Circumference [HC], Systolic Blood Pressure [SBP], Diastolic Blood Pressure), chronic disease history (Diabetes Hx), and laboratory test indicators: Absolute Monocyte Count (AMC), Platelet-Large Cell Ratio (P-LCR), Albumin/Globulin Ratio (A/G), Mean Corpuscular Hemoglobin (MCH), Albumin (ALB), Direct Bilirubin (DBIL), and Triglycerides (TG). The psychological dimension was assessed using standardized scales: the Patient Health Questionnaire-9 (PHQ-9) was used to screen for and classify Depression Severity (39), and the Self-Rating Anxiety Scale (SAS) was used to assess anxiety levels (40). The behavioral dimension comprehensively recorded lifestyle habits, including Smoking Hx, Sleep Duration, physical activity level (Moderate PA), sugary beverage intake, and dietary patterns (including frequencies of staple food, vegetable, fruit, dairy product, and milk tea consumption). To align with the local cultural context, subjective saltiness perception of specific foods—pilaf (Sodium Pilaf), naan bread, and pickled foods (Sodium Pickled Food)—was also investigated. The social dimension encompassed Marital Status, Education, Occupation, Insurance Type, annual Household Income, and the Lubben Social Network Scale was utilized to assess social isolation (41), objectively reflecting the level of social support.

### Data preprocessing and feature selection

The primary objective of data preprocessing was to enhance data quality and optimize model performance. In this study, missing values were handled using deletion and imputation methods: participants with > 20% missing data were excluded first, followed by multiple imputation for the remaining missing values. Categorical variables were converted into factor types to ensure correct recognition and processing by the models. After partitioning the data into training and validation sets (7:3 ratio), numerical variables within the training set were standardized (mean = 0, standard deviation = 1) to eliminate the influence of differing scales on model fitting. The standardization parameters derived from the training set were subsequently applied to the validation set to maintain consistency in data preprocessing. These preprocessing procedures were executed exclusively within training subsets to eliminate data leakage risks. Missing value imputation and Z-score standardization were calculated separately for each cross-validation fold using only training subset statistics. All follow-up LASSO feature screening, SMOTE oversampling, hyperparameter optimization and model fitting were completed via 10-fold cross-validation limited to training data; the validation set was completely isolated and only used for final internal validation assessment.

To enhance model recognition performance, this study employed a combined strategy of embedded feature selection and sequential forward selection (42). The specific procedure was as follows: (1) Initially, LASSO regression was utilized to screen for feature variables associated with MoCA-defined possible MCI from the initial set of 33 clinical features, accomplishing initial dimensionality reduction. (2) The selected features were then ranked according to their importance as determined by the XGBoost algorithm and iteratively incorporated into an XGBoost classifier using sequential forward selection. (3) Model performance changes were evaluated with each iterative inclusion of a new feature. (4) Based on the performance evaluation results, the optimal feature subset was identified for constructing the final screening model. This method, combining feature importance ranking with performance validation, ensured the reliability and efficiency of feature selection.

### Machine learning models

Given that preliminary data analysis indicated a significant class imbalance between MoCA-defined possible MCI-positive and MoCA-defined possible MCI-negative cases, which could potentially impact the training effectiveness and generalization capability of machine learning models, the Synthetic Minority Over-sampling Technique (SMOTE) was applied to optimize data distribution. This technique operates on the principle of generating synthetic samples for the minority class through interpolation within the feature space based on k-nearest neighbors, thereby balancing class distribution (43).

The dataset was randomly allocated to a training set (70%) for model development and an internal validation set (30%) for model evaluation. To prevent model overfitting, 10-fold cross-validation was implemented. Six representative machine learning algorithms were selected for constructing screening models: Logistic Regression (LR), Adaptive Boosting (AdaBoost), Multilayer Perceptron (MLP), Naïve Bayes (NB), Extreme Gradient Boosting (XGBoost), and Light Gradient Boosting Machine (LightGBM). Grid search combined with 10-fold cross-validation was conducted solely on the training set for hyperparameter optimization. A fixed random seed of 123 was used throughout all analyses for procedural reproducibility. All statistical analyses were performed using R 4.4.3. Nested cross-validation was not applied, given the limited overall sample size; we adopted single-layer 10-fold cross-validation for parameter tuning and reserved an independent validation cohort for final model assessment. Full tuning grids and optimal hyperparameters are summarized in Supplementary Table S2.

### Model performance and evaluation

Model performance was assessed using the validation set. Internal validation performance was evaluated by calculating the mean Area Under the Receiver Operating Characteristic Curve (AUROC) and its 95% Confidence Interval (CI). The optimal machine learning screening model was selected based on AUC value. Model performance was further quantified using the following metrics: Sensitivity, Specificity, Accuracy, Precision, and F1-Score. In addition, model calibration was quantified via Brier score, calibration slope and calibration intercept. Calibration curves mapping predicted risk against observed outcomes were generated for the training and validation datasets of all algorithms. Decision curve analysis (DCA) was conducted for each model to compare net clinical benefit against the “screen all” and “screen none” reference strategies.

### Model interpretation

To enhance the transparency and interpretability of the machine learning screening model, SHapley Additive explanations (SHAP) was employed for feature importance analysis (44). This method, rooted in game theory, is a commonly used approach for interpreting the outputs of machine learning models. Its core principle involves quantifying the contribution of individual features by evaluating their impact during the collaborative recognition process. Within the SHAP framework, each feature is assigned an importance value (i.e., SHAP value), enabling a fair distribution of recognition influence among different variables. A higher mean absolute SHAP value indicates greater importance of the feature for MoCA-defined possible MCI identification. Positive SHAP values indicate an association of the corresponding feature with MoCA-defined possible MCI positivity, negative SHAP values suggest an association with MoCA-defined possible MCI negativity, and SHAP values close to zero imply minimal association with the recognition outcome. Additionally, to explore potential non-linear relationships between feature variables and MoCA-defined possible MCI, Restricted Cubic Splines (RCS) were employed to fit dose–response curves (45). This method allows for flexible characterization of complex associations between variables and outcomes by placing knots within the range of the continuous variable and fitting piecewise cubic polynomials.

### Statistical analysis

The Kolmogorov–Smirnov (K-S) test was used to assess the normality of data distribution. Normally distributed continuous variables were summarized as mean ± standard deviation (SD) and between-group comparisons were performed using the t-test. Non-normally distributed continuous variables were presented as median (*P*_25_, *P*_75_), and the Mann–Whitney U test was used for group comparisons. Categorical variables were presented as frequencies (percentages), and the Chi-square test or Fisher’s exact test was used for between-group comparisons. All statistical tests were two-sided, and a two-sided *P* < 0.05 was considered statistically significant. All graph plotting and statistical analyses were performed using R version 4.4.3.

## Results

### Characteristics

A total of 708 participants were enrolled in this study. Among them, 497 were allocated to the training set, including 368 individuals with MoCA-defined possible MCI. The validation set comprised 211 participants, of whom 157 were classified as having MoCA-defined possible MCI. The two groups were well-balanced in terms of demographic, clinical, and behavioral characteristics, with only TG levels (*p* = 0.032) showing a statistically significant difference between the training and validation sets (Supplementary Table S1).

### Preprocessing of data and screening of variables

**Figure 1:**
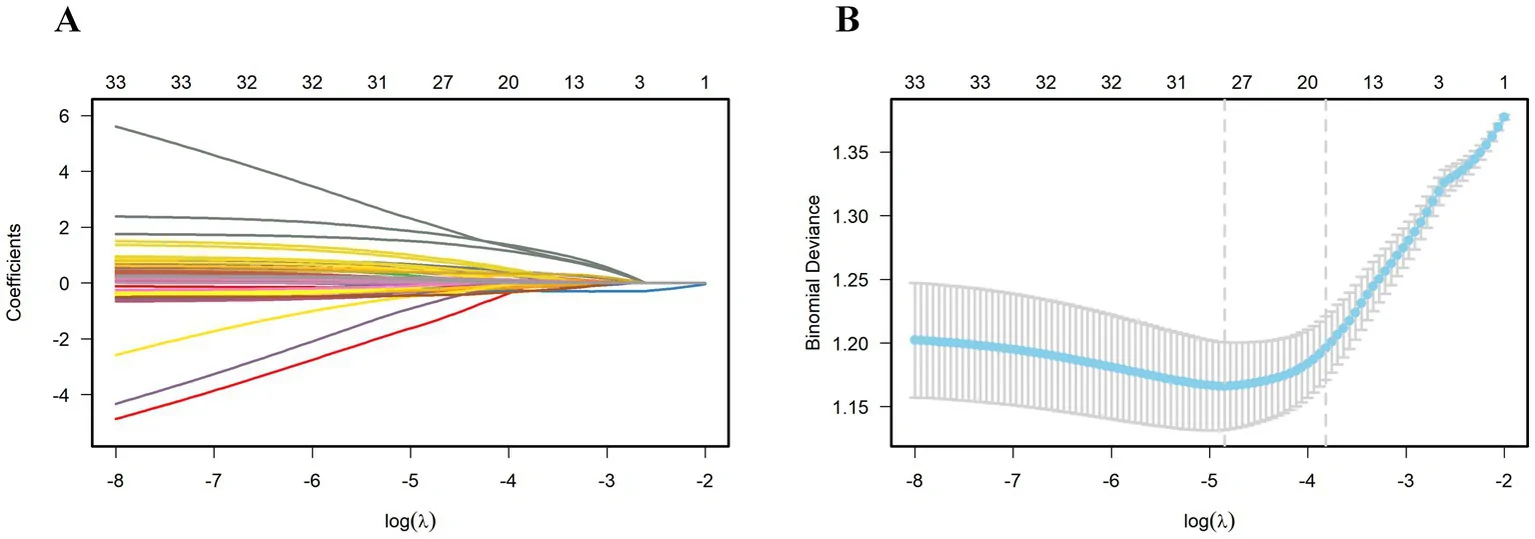
Feature selection via LASSO regression. **(A)** The coefficient profile plot produced versus the log (*λ*). **(B)** The adjustment parameter (*λ*) was selected using 10-fold cross-validation in the LASSO model. The binomial deviance curve was plotted against log (*λ*). The dotted vertical lines indicated the optimal predictors using the minimum criteria (min. criteria) and the 1 standard error (SE) of the minimum criteria (1-SE criteria).

**Figure 2:**
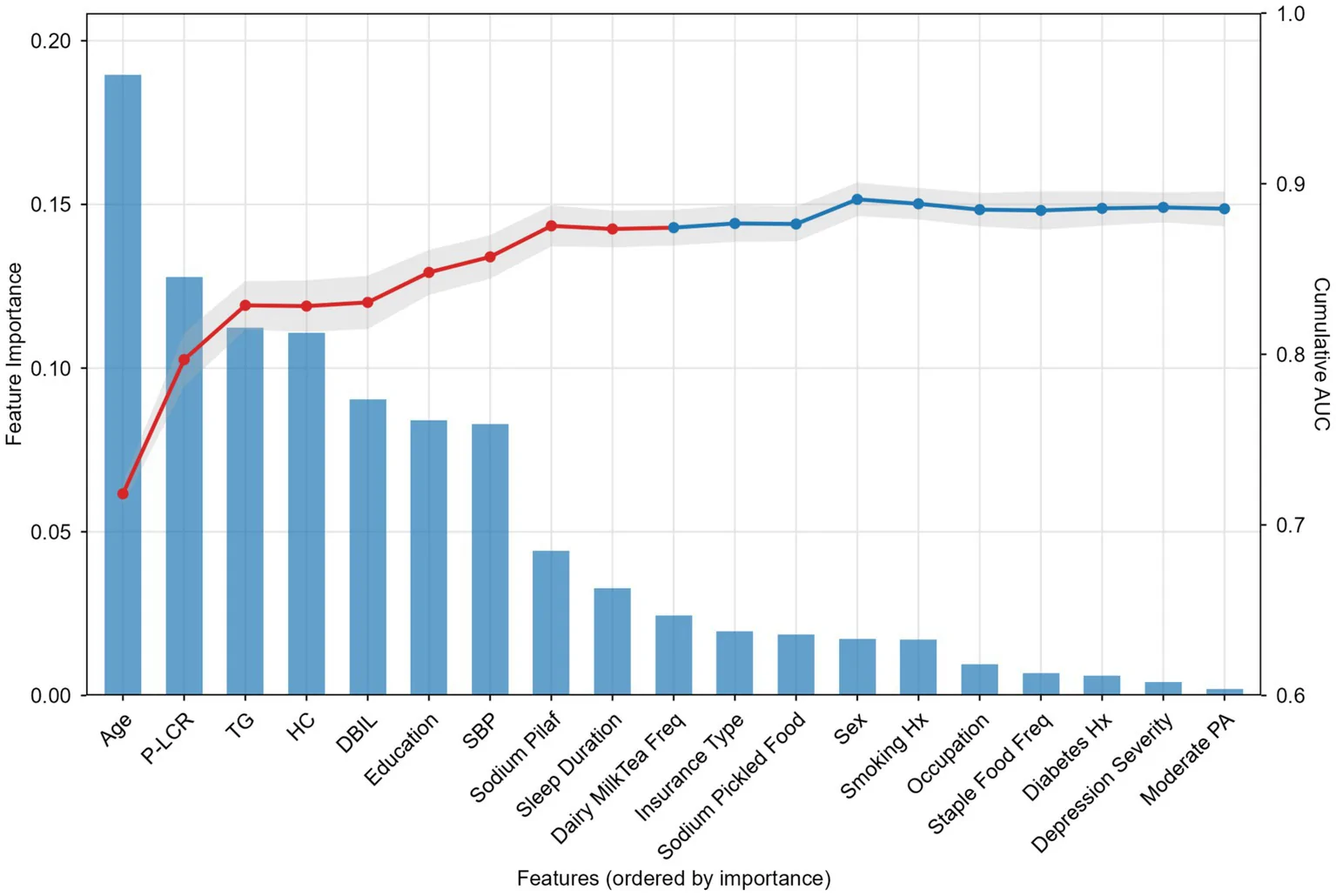
Feature importance and cumulative AUC curve for predictor screening based on XGBoost algorithm. The horizontal axis represents the number of predictor variables included in turn according to the feature importance ranking, and the vertical axis represents the cumulative AUC value of the model. The curve tends to be stable when 9 variables are included, indicating that the optimal feature subset is determined with 9 core predictors.

This study implemented a two-step feature selection process using LASSO regression and the XGBoost algorithm. Initially, 33 features were standardized. The optimal regularization parameter *λ* for the LASSO model was determined via 10-fold cross-validation, selecting lambda.1se as the optimal value. This step identified 19 potential feature variables with non-zero coefficients, as illustrated in Figure 1. Subsequently, features were ranked by importance calculated using the XGBoost algorithm. Variables were progressively incorporated into the model based on the trend of cumulative AUC change. It was observed that model performance plateaued after the inclusion of the top 9 features, with no significant gain from adding further variables. Consequently, these 9 features were selected as the core predictor set for MoCA-defined possible MCI: Age, P-LCR, TG, HC, DBIL, Education, SBP, Sodium Pilaf, and Sleep Duration. This process is detailed in Figure 2.

The Synthetic Minority Over-sampling Technique (SMOTE) was applied to the training set to address class imbalance. Following over-sampling, the sample sizes for both the MoCA-defined possible MCI and non-MoCA-defined possible MCI groups in the training set were balanced at 368 cases each.

### Model performance and comparison

Using the nine selected variables (Age, P-LCR, TG, HC, DBIL, Education, SBP, Sodium Pilaf, Sleep Duration) as features, six machine learning algorithms—Logistic Regression, AdaBoost, MLP, Naïve Bayes, XGBoost, and LightGBM—were employed to construct MoCA-defined possible MCI screening models. Model performance was evaluated using 10-fold cross-validation, with detailed results presented in Table 1.

**Table 1:** Performance comparison of six machine learning models in the validation set.

Model	AUC (95% CI)	Accuracy	Sensitivity	Specificity	Precision	F1
Logistic	0.726 (0.654–0.797)	0.649	0.650	0.648	0.843	0.734
AdaBoost	0.746 (0.674–0.818)	0.654	0.631	0.722	0.868	0.731
MLP	0.717 (0.644–0.790)	0.640	0.631	0.667	0.846	0.723
Naïve Bayes	0.722 (0.648–0.797)	0.659	0.669	0.630	0.840	0.745
XGBoost	0.747 (0.673–0.820)	0.692	0.688	0.704	0.871	0.769
LightGBM	0.729 (0.656–0.802)	0.668	0.650	0.722	0.872	0.745

**Figure 3:**
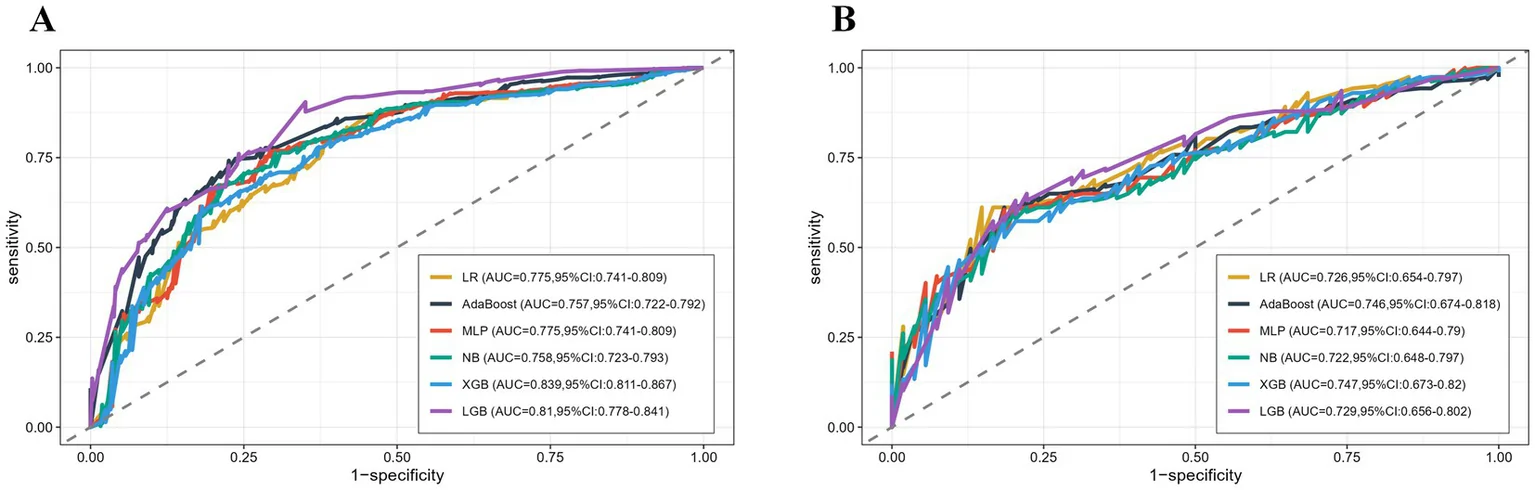
Receiver-operating characteristic curve (ROC) for different machine learning models in the training set **(A)** and validation set **(B)**.

In the validation set, the Area Under the Curve (AUC) values for the models, ranked from highest to lowest, were as follows: XGBoost, AdaBoost, Naïve Bayes, LightGBM, Logistic Regression, and MLP. The XGBoost model exhibited the best performance, achieving a validation set AUC of 0.747. In the training set, XGBoost demonstrated an AUC of 0.839, accuracy of 0.746, sensitivity of 0.731, specificity of 0.761, precision of 0.754, and an F1 score of 0.742, indicating strong classification performance and good model fit. The Receiver Operating Characteristic curves for all models in both the training and validation sets are depicted in Figure 3.

**Figure 4:**
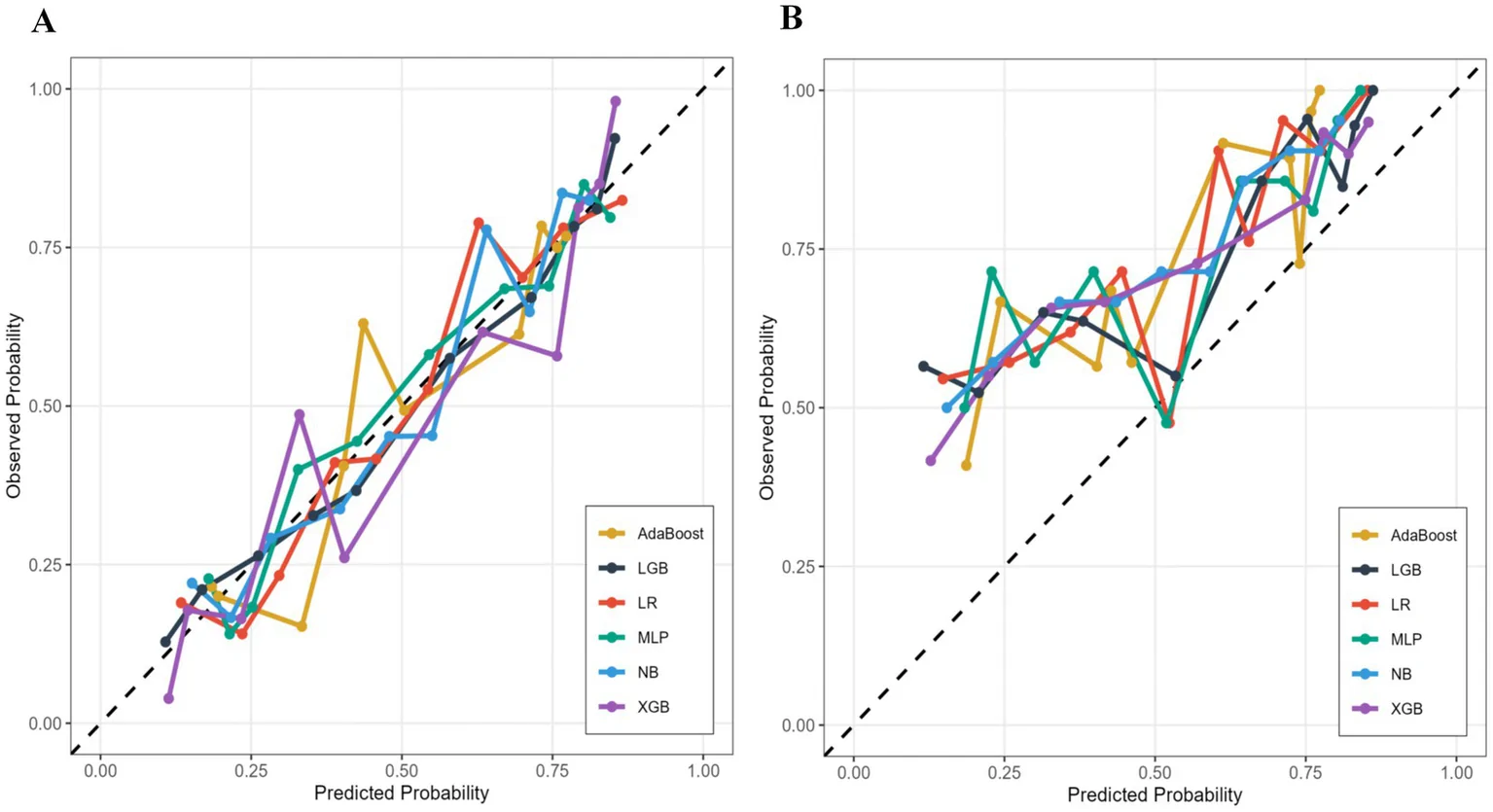
Calibration curves for six machine learning models after Platt scaling. The x-axis represents the predicted probability of cognitive impairment, the y-axis represents the observed probability, and the dashed diagonal line indicates perfect calibration. **(A)** Calibration curves in the training set. **(B)** Calibration curves in the validation set.

After Platt scaling probability calibration, the XGBoost model yielded a Brier score of 0.208, a calibration slope of 0.720, and a calibration intercept of 1.07 in the validation set (Supplementary Table S3). Calibration curves for the training and validation sets are shown in Figures 4A,B, respectively. Supplementary Figure S2 presents the decision curve analysis, which showed positive net clinical benefit within the threshold range of 0.1 to 0.8.

### Model interpretation

**Figure 5:**
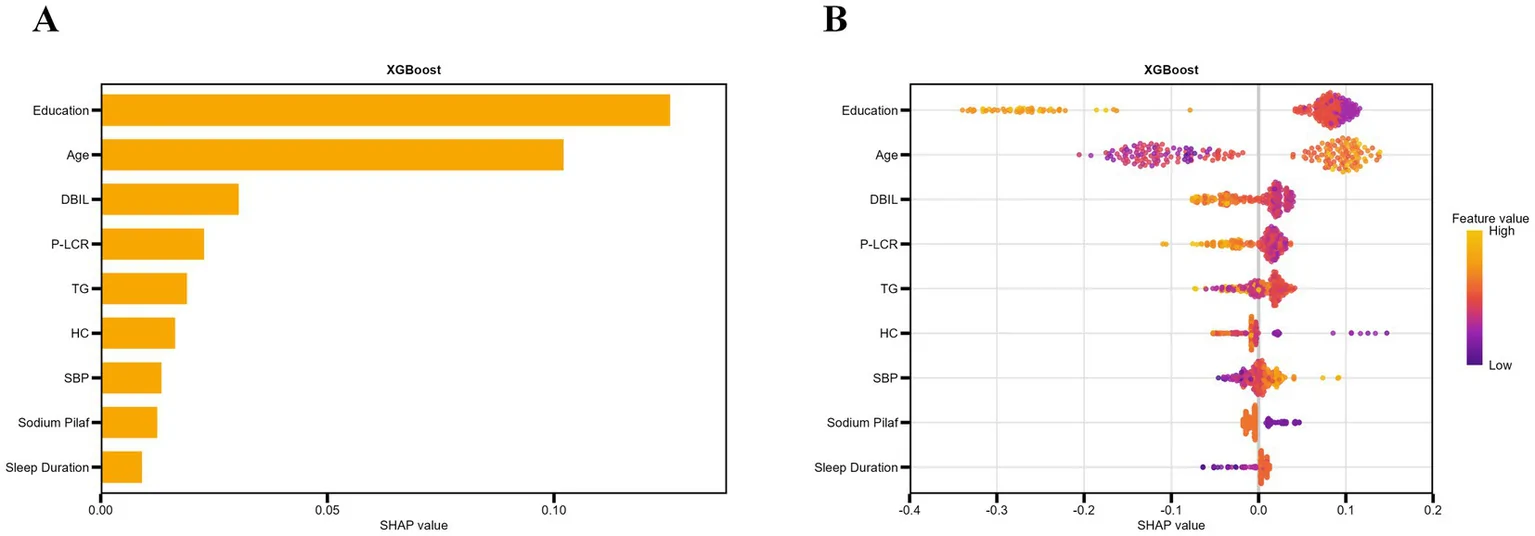
Interpretation of the XGBoost model based on SHAP. The x-axis denotes the contribution of each indicator to the prediction model. The y-axis indicates the characteristic value of each indicator, with all features presented. **(A)** Bar plot of feature importance, displaying the average SHAP values for each feature. **(B)** Summary plots showing the impact of each feature on model output. A positive value would augment the predicted result, whereas a negative value would reduce it.The yellow dots represent high characteristic values, which indicate high risk, while the purple dots represent low characteristic values, which indicate low risk.

Within an interpretable machine learning framework, the SHAP method was applied to the XGBoost model to quantify the contribution of each input feature to the model’s output. The SHAP feature importance bar plot, with mean absolute SHAP value on the horizontal axis and feature variables on the vertical axis (Figure 5A), indicated that based on SHAP value ranking, Education, Age, and DBIL were the top three most influential variables affecting MoCA-defined possible MCI identification. These were followed by P-LCR, TG, HC, SBP, Sodium Pilaf, and Sleep Duration.

In the SHAP beeswarm plot (Figure 5B), the horizontal axis represents SHAP values, while the vertical axis lists variables in descending order of importance. The position of data points along the horizontal axis reflects the direction and magnitude of each feature’s contribution to MoCA-defined possible MCI prediction, with the color gradient indicating feature value (yellow for high values, purple for low values). Points distributed toward the positive side of the horizontal axis indicate that the corresponding feature value is associated with MoCA-defined possible MCI positivity, whereas points toward the negative side suggest an association with MoCA-defined possible MCI negativity. The results in Figure 5B show that higher values for Age and SBP were associated with a tendency toward positive SHAP values, indicating an increased probability of MoCA-defined possible MCI classification. Conversely, higher values for Education, DBIL, P-LCR, and TG were associated with a tendency toward negative SHAP values, significantly decreasing the probability of MoCA-defined possible MCI classification.

### Restricted cubic splines

**Figure 6:**
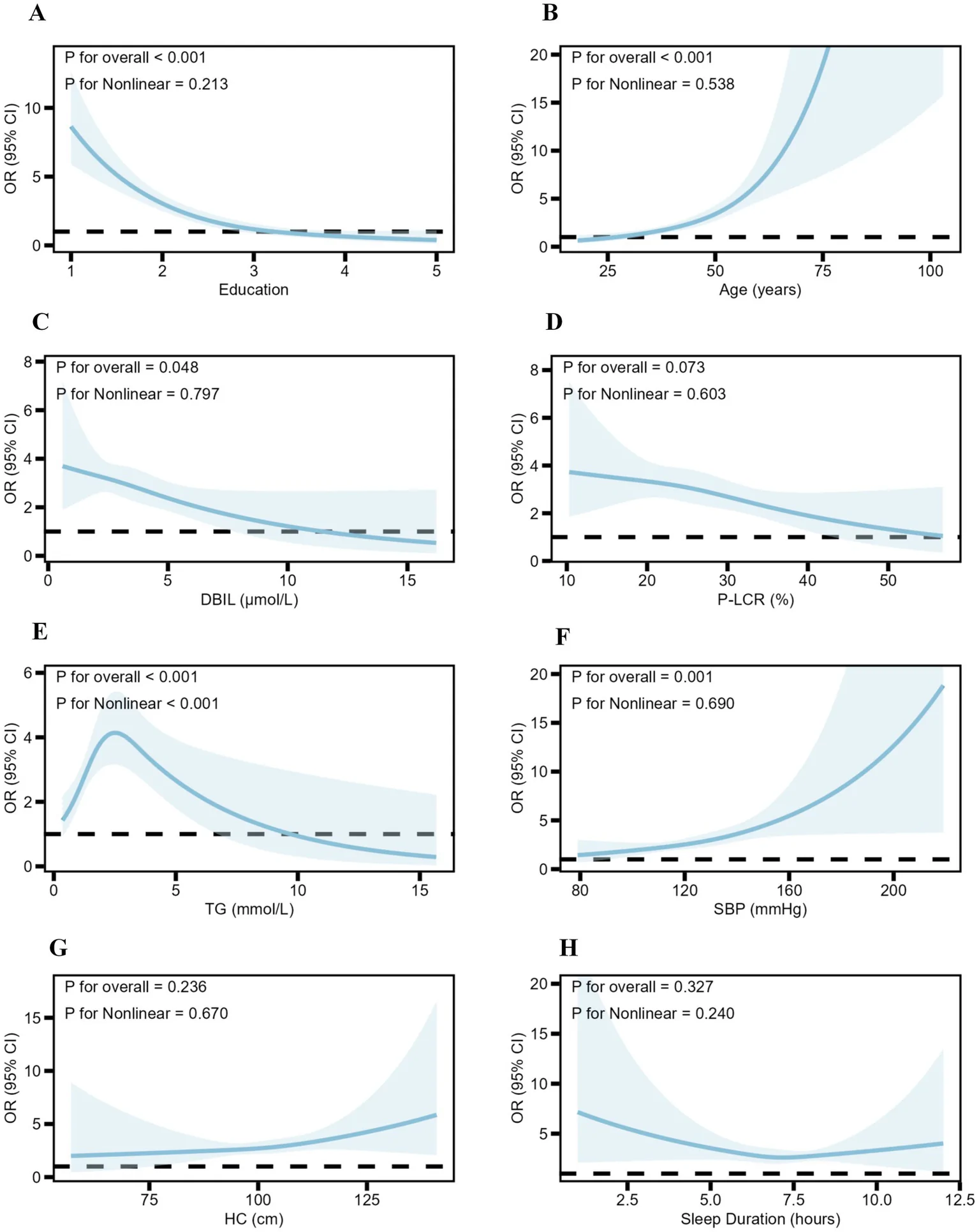
Restricted cubic spline curves for the dose–response relationship between continuous predictors and the risk of cognitive impairment. The solid line represents the fitted risk trend of cognitive impairment, the shaded area is the 95% confidence interval, and the x-axis is the normalized value of each continuous predictor. The model reveals the linear or non-linear correlation between each indicator and the risk of cognitive impairment after adjusting for confounding factors.

This study employed Restricted Cubic Spline (RCS) models to systematically evaluate the dose–response relationships between eight clinical indicators and MoCA-defined possible MCI risk (Figure 6). The analysis revealed distinct association patterns across indicators. Linear correlations with MoCA-defined possible MCI risk were observed for Education, Age, DBIL, and SBP (overall association test *p* < 0.05, non-linearity test *p* > 0.05). In contrast, TG exhibited a non-linear association with MoCA-defined possible MCI risk (overall *p* < 0.001, non-linear *p* < 0.001), with its dose–response curve displaying a complex non-linear trend. Conversely, no statistically significant overall association or non-linear relationship was observed for P-LCR, HC, or Sleep Duration with MoCA-defined possible MCI risk, suggesting the absence of a clear dose–response relationship for these three variables.

### Clinical translation subsection

To facilitate primary care adoption, we established a threshold-based risk stratification strategy derived from calibrated XGBoost predicted probabilities. The optimal cutoff maximizing the Youden index was 0.538; a provisional rounded threshold of 0.50 is suggested as an exploratory cutoff for daily routine screening in similar populations, derived solely from internal validation and requiring prospective external validation before clinical implementation. A higher-specificity provisional threshold of 0.670 is proposed as an exploratory cutoff for specialist referral consideration, derived solely from internal validation and requiring prospective external validation before clinical implementation. Complete multi-threshold performance metrics are listed in Supplementary Table S3. The proposed simplified 20-point manual risk scoring system is provided as an exploratory auxiliary tool for rough screening in settings without computational support; it requires independent external and prospective validation before any clinical use. A standardized five-step primary care implementation workflow is summarized in Supplementary Table S5 and Supplementary Figure S3. All thresholds, risk stratification schemes, and the simplified scoring system presented herein are exploratory and derived exclusively from internal validation within a single cohort. They are not intended as definitive clinical recommendations and require prospective external validation in diverse populations before any clinical deployment. The simplified score only serves as an auxiliary rough screening tool without computing equipment; the full calibrated XGBoost model is recommended for accurate probability prediction.

## Discussion

Cognitive impairment continues to impose a growing global public health burden (46), and its early identification and intervention are particularly critical in resource-limited settings. Although evidence has explored MoCA-defined possible MCI risk factors across physiological, psychological, behavioral, and social domains, the systematic integration and application of these factors within screening models remain inadequate. This study applies an established machine learning pipeline to systematically evaluate the combined predictive value of nine factors for identifying MoCA-defined possible MCI in a rural Xinjiang population. The primary innovation of this work lies in its localization and preliminary implementation framework for resource-limited primary care settings, providing a conceptual pathway that can be adapted to similar populations with distinct cultural and dietary characteristics. A risk identification model was constructed using LASSO regression and the XGBoost algorithm, achieving an AUC of 0.747 in the validation set, demonstrating moderate discriminative ability that is clinically acceptable for a preliminary screening tool in resource-limited settings. SHAP-based interpretability analysis revealed that Education, Age, and DBIL were the top three contributing features. Higher values for Education and DBIL corresponded to negative SHAP contributions, whereas higher Age corresponded to a positive SHAP contribution. These patterns are consistent with the cognitive reserve hypothesis (46), whereby higher educational attainment may delay the clinical manifestation of cognitive dysfunction. The positive contribution of Age aligns with established age-related cognitive trajectories (47) and the negative contribution of DBIL is consistent with bilirubin’s antioxidant properties, which may protect against oxidative stress-induced neuronal damage (48). These interpretations, while grounded in existing biological literature, remain associative and require longitudinal validation.

To ensure an analytical framework that is robust and clinically applicable, this study, based on cross-sectional data from 708 residents of rural Xinjiang, established an MoCA-defined possible MCI risk identification system encompassing physiological, psychological, behavioral, and social dimensions. All participants were classified for cognitive status using the MoCA, and strict inclusion and exclusion criteria were applied to control for confounding bias. The training and validation sets were well-balanced regarding demographic and clinical characteristics, with only TG levels showing a statistical difference between groups (*p* = 0.032). During feature selection, 19 candidate variables with non-zero coefficients were initially identified via LASSO regression, and the top 9 core predictors were subsequently determined through XGBoost feature importance ranking. Among the six algorithms compared (Logistic Regression, AdaBoost, MLP, Naïve Bayes, XGBoost, and LightGBM), the XGBoost model performed optimally, yielding a validation‑set AUC of 0.747 (95% CI: 0.673–0.820), sensitivity of 0.688, and specificity of 0.704. While the validation AUC of 0.747 indicates moderate discrimination, this level of performance is generally considered clinically acceptable for resource-limited primary care screening relying only on routine variables. This performance is comparable to some screening tools based on Western populations (49) and AI-driven models utilizing electronic health records (50). Although AdaBoost achieved a nearly identical AUC (0.746), XGBoost demonstrated a substantially higher F1-score and greater net clinical benefit across common screening thresholds, indicating better balanced classification and greater practical value for primary care applications. We suggest that this performance profile may stem primarily from the localization of features specific to the surveyed population’s socio-cultural background, dietary structure, and comorbidity patterns. Specifically, the inclusion of dietary factors and metabolic markers in the model more accurately reflects the actual exposure characteristics of this population, thereby enhancing the model’s applicability and predictive accuracy in primary care settings and providing a viable pathway for early risk mitigation in resource-limited areas.

We acknowledge that the observed prevalence of MoCA-defined possible MCI in our sample (approximately 74%) is substantially higher than the national estimate of 20.8% (35). We believe this primarily reflects the low educational attainment of our rural population—the MoCA is known to be highly sensitive to educational level, with lower education associated with higher false-positive rates (51). In our study, the majority of participants had only primary school education or were illiterate, and many struggled with the visuospatial/executive and delayed recall tasks, which are particularly affected by educational background. Moreover, the standard +1 point adjustment for ≤ 12 years of education may still be insufficient to fully compensate for the educational disadvantage in populations with very low literacy levels. These findings underscore that the MoCA is a screening tool; participants identified as having MoCA-defined possible MCI require confirmatory comprehensive neuropsychological assessment. We have therefore consistently used the term ‘MoCA-defined possible MCI’ throughout this manuscript.

Based on SHAP analysis, the ranked importance of features for MoCA-defined possible MCI identification in rural Xinjiang was Education, Age, DBIL, P-LCR, TG, and SBP. This ranking reflects the model’s reliance on these features during decision-making, indicating particular sensitivity to cognitive reserve insufficiency, age-related changes, and metabolic-antioxidant status. Education, as the feature with the highest contribution, exhibited a clear negative correlation with MoCA-defined possible MCI, meaning that longer education duration was associated with a lower probability of being classified as having MoCA-defined possible MCI. This finding aligns strongly with Stern’s cognitive reserve hypothesis (52)—higher educational attainment enhances neuroplasticity and synaptic reserve, thereby delaying the clinical manifestation of cognitive dysfunction. A recent review encompassing global cohorts confirmed that each additional year of education is associated with an approximately 11% reduction in dementia incidence risk (46) and individuals with higher education levels can maintain better cognitive function even in the presence of considerable brain pathology. Age, as the second most important feature, demonstrated a positive contribution consistent with MoCA-defined possible MCI being an age-related condition (26) and aligning with findings from multiple global cohort studies (53). DBIL ranked third in importance. SHAP analysis indicated that higher DBIL levels were associated with a lower probability of the model screening for MoCA-defined possible MCI, suggesting that, within the model’s decision-making context for this population, higher DBIL levels exert a protective contribution. The observed negative association between DBIL and MoCA-defined possible MCI is consistent with the neuroprotective properties of bilirubin, which may act through scavenging reactive oxygen species and inhibiting inflammatory cell recruitment. This aligns with findings from the UK Biobank cohort (54). While our observation of bilirubin‑related protection agrees with the Korean hemodialysis patient study (55), that study reported that when DBIL exceeds a certain threshold, the protective effect may plateau or even reverse, suggesting that maintaining optimal bilirubin levels may be important for cognitive health. These interpretations remain associative and require longitudinal validation. P-LCR and TG also showed negative SHAP contributions, meaning higher levels of P-LCR and TG were associated with a lower probability of MoCA-defined possible MCI identification in this model. This directional finding is consistent with the hypothesis that, within this specific population, moderately elevated P-LCR might reflect healthy platelet turnover and renewal activity rather than purely pathological activation. This contrasts with prior observations linking P-LCR positively with neurodegenerative pathology burden (56), a discrepancy that may reflect ethnic or metabolic background differences. These interpretations remain speculative given the cross-sectional design. The negative contribution of TG is consistent with the unique metabolic profile of this population, where very low TG levels could indicate malnutrition or chronic disease states, while the non-linear dose–response curve suggests complex metabolic dynamics rather than a simple linear benefit across the TG spectrum. This pattern is consistent with findings from a prospective cohort study in community-dwelling older adults (57). However, this interpretation remains associative and requires confirmation through longitudinal designs. The positive SHAP contribution of SBP further reinforces the foundational role of vascular risk factors. In summary, SHAP analysis indicates that MoCA-defined possible MCI in this region is primarily driven by cognitive reserve depletion and age-related changes, whereas an optimal metabolic-antioxidant status appears to confer a protective effect.

Restricted cubic spline regression further evaluated the association patterns between each feature and the model’s predicted probability. The results showed a significant linear negative correlation between Education and predicted probability (overall *p* < 0.05, non-linear *p* > 0.05), supporting the continuous nature assumption of cognitive reserve theory—that the cumulative protective effect of education years exhibits no obvious saturation threshold (46). Furthermore, highly educated individuals may demonstrate greater compensatory capacity against white matter lesions, potentially maintaining better cognitive function even in the presence of considerable brain pathology (58). Age exhibited a linear positive correlation with MoCA-defined possible MCI, consistent with the epidemiological characteristics of MoCA-defined possible MCI as an age-related condition and mirroring age-related cognitive trajectories observed in the Rotterdam Study (59). DBIL showed a significant linear negative correlation (overall *p* < 0.05), consistent with the antioxidant protective effect of bilirubin observed in the Korean hemodialysis patient cohort (55)The neuroprotective properties of bilirubin may act through scavenging reactive oxygen species and inhibiting inflammatory cell aggregation (60). SBP was linearly and positively associated with MoCA-defined possible MCI (overall p < 0.05, non-linear *p* > 0.05), consistent with findings from the Northern Manhattan Study (NOMAS), which observed a linear inverse relationship between SBP and processing speed/visuomotor integration (61). This is consistent with vascular pathway hypotheses, whereby elevated blood pressure may contribute to cognitive impairment through accelerating cerebral small vessel disease and promoting blood–brain barrier disruption (62). In contrast, TG exhibited a significant non-linear association (overall *p* < 0.001, non-linear *p* < 0.001), with its dose–response curve displaying a complex trend. This partially aligns with results from the ARIC study (63), which found no significant linear association between midlife dyslipidemia and late-life cognitive function, though the non-linear pattern observed in our study may reflect the unique dietary lipid intake profile of the Xinjiang population. The significant non-linear association between TG and MoCA-defined possible MCI may reflect complex metabolic pathways involving insulin resistance, cerebrovascular endothelial dysfunction, and inflammatory cytokine release (64). The heterogeneity observed in these dose–response relationships suggests that MoCA-defined possible MCI prevention interventions require differentiated strategies: for linear protective factors like Education, the emphasis should be on the long-term cognitive benefits of lifelong educational investment; for linear risk factors like SBP, strict blood pressure control targets are necessary; and for non-linear factors like TG, identifying an optimal metabolic range rather than pursuing a single lipid-lowering target is paramount. The linear and non-linear association patterns revealed in this study provide a new evidence base for implementing precise MoCA-defined possible MCI identification and risk stratification in resource-limited settings.

### Limitations

This study has several limitations. First, this study adopted a single-center cross-sectional design. Although Tumxuk City was selected as a typical agricultural region and five villages were randomly chosen using a random number table, the findings cannot be generalized to all rural residents across Xinjiang given the region’s vast territory and ethnic diversity, and are only applicable to populations with comparable demographic and lifestyle profiles. Furthermore, predictors such as Sodium Pilaf reflect local dietary habits specific to Xinjiang and may not transfer directly to populations with different cultural practices. For broader application, culturally specific variables could be replaced with locally relevant dietary proxies during model recalibration, while core predictors including Age, Education, SBP, DBIL, and TG are likely transferable across settings. The model may be particularly suitable for populations with similar dietary and cultural characteristics, and model recalibration in external populations is recommended before implementation in other regions.

## Conclusion

This study integrated multidimensional data from residents in rural primary care settings. Through variable selection via LASSO regression and the construction of multiple machine learning models, XGBoost was identified as the optimal screening model. Requiring only nine easily obtainable indicators at the primary care level, this model demonstrates moderate discriminative performance and useful clinical interpretability, offering a preliminary tool for the early identification of MoCA-defined possible MCI in resource-limited settings. The findings not only contribute to advancing precise prevention and control of MoCA-defined possible MCI among rural residents facing healthcare resource constraints but also provide a potential methodological reference for conducting localized screening research in similar regions, though prospective external validation is essential.

## Data Availability

The original contributions presented in the study are included in the article/Supplementary material, further inquiries can be directed to the corresponding authors.

## Glossary

MCI: Mild cognitive impairment
MoCA: Montreal Cognitive Assessment
LR: Logistic Regression
AdaBoost: Adaptive Boosting
MLP: Multilayer Perceptron
NB: Naïve Bayes
XGBoost: Extreme Gradient Boosting
LightGBM: Light Gradient Boosting Machine
AUC: Area Under the Curve
SHAP: SHapley Additive explanations
RCS: Restricted Cubic Splines
LASSO: Least Absolute Shrinkage and Selection Operator
SBP: Systolic Blood Pressure
DBP: Diastolic Blood Pressure
HC: Hip Circumference
WC: Waist Circumference
P-LCR: Platelet-Large Cell Ratio
DBIL: Direct Bilirubin
TG: Triglycerides
AMC: Absolute Monocyte Count
A/G: Albumin/Globulin Ratio
MCH: Mean Corpuscular Hemoglobin
ALB: Albumin
PA: Physical Activity
Hx: History
