# Supplementary material for: An interpretable machine learning screening model for MoCA-defined possible mild cognitive impairment in rural Xinjiang: a preliminary framework for resource-limited primary care
Source: Front Public Health. 2026 Aug 26;14:1854956. doi: 10.3389/fpubh.2026.1854956 (PMC13553415; doi:10.3389/fpubh.2026.1854956)
Supplement: Supplementary file 1 [file Table_1.DOCX]

**Supplementary Materials**

**Supplementary Tables**

**Supplementary Table S1. Baseline characteristics of participants in the training and validation sets**

| **Variables** | **Train data (n = 497)** | **Validation set (n = 211)** | ***P*** |
| --- | --- | --- | --- |
| Depression Severity, n (%) |  |  | 0.994 |
| No Depression | 355 (71.4%) | 165 (78.2%) |  |
| Mild Depression | 103 (20.7%) | 35 (16.6%) |  |
| Moderate Depression | 32 (6.4%) | 9 (4.3%) |  |
| Severe Depression | 7 (1.4%) | 2 (0.9%) |  |
| Social Isolation Severity, n (%) |  |  | 0.999 |
| No | 395 (79.5%) | 167 (79.1%) |  |
| Yes | 102 (20.5%) | 44 (20.9%) |  |
| Anxiety Severity, n (%) |  |  | 0.662 |
| No Anxiety | 354 (71.2%) | 157 (74.4%) |  |
| Mild Anxiety | 122 (24.5%) | 49 (23.2%) |  |
| Moderate Anxiety | 15 (3.0%) | 3 (1.4%) |  |
| Severe Anxiety | 6 (1.2%) | 2 (0.9%) |  |
| Sex, n (%) |  |  | 0.830 |
| Male | 206 (41.4%) | 90 (42.7%) |  |
| Female | 291 (58.6%) | 121 (57.3%) |  |
| Age (years) | 49.0 (41.0, 55.0) | 47.0 (41.0, 56.0) | 0.427 |
| SBP (mmHg) | 126.0 (113.0, 139.0) | 124.0 (113.0, 136.0) | 0.301 |
| DBP (mmHg) | 76.0 (69.0, 83.0) | 74.0 (68.0, 82.0) | 0.322 |
| WC (cm) | 96.4 ± 13.8 | 95.1 ± 13.0 | 0.225 |
| HC (cm) | 102.0 (96.0, 108.0) | 101.0 (97.0, 108.5) | 0.905 |
| Marital Status, n (%) |  |  | 0.389 |
| Married | 466 (93.8%) | 202 (95.7%) |  |
| Unmarried/Divorced/Widowed | 31 (6.2%) | 9 (4.3%) |  |
| Education, n (%) |  |  | 0.729 |
| Illiterate/Semi illiterate | 157 (31.6%) | 61 (28.9%) |  |
| Primary School | 239 (48.1%) | 101 (47.9%) |  |
| Junior High School | 72 (14.5%) | 37 (17.5%) |  |
| Senior High School | 23 (4.6%) | 8 (3.8%) |  |
| College | 6 (1.2%) | 4 (1.9%) |  |
| Occupation, n (%) |  |  | 0.254 |
| Farmer | 314 (63.2%) | 131 (62.1%) |  |
| Worker | 60 (12.1%) | 19 (9.0%) |  |
| Professional and Technical Personnel | 14 (2.8%) | 10 (4.7%) |  |
| Commercial/Service Industry Employee | 40 (8.0%) | 24 (11.4%) |  |
| General Staff | 6 (1.2%) | 1 (0.5%) |  |
| Cadre | 0 (0.0%) | 1 (0.5%) |  |
| Others | 63 (12.7%) | 25 (11.8%) |  |
| Insurance Type, n (%) |  |  | 0.920 |
| Urban Rural Resident Medical Insurance | 76 (15.3%) | 30 (14.2%) |  |
| Urban Resident Medical Insurance | 397 (79.9%) | 170 (80.6%) |  |
| New Rural Cooperative Medical Insurance | 24 (4.8%) | 11 (5.2%) |  |
| Diabetes Hx, n (%) |  |  | 0.097 |
| No | 451 (90.7%) | 200 (94.8%) |  |
| Yes | 46 (9.3%) | 11 (5.2%) |  |
| Moderate PA, n (%) |  |  | 0.999 |
| No | 461 (92.8%) | 196 (92.9%) |  |
| Yes | 36 (7.2%) | 15 (7.1%) |  |
| Sugary Food/SSB Intake, n (%) |  |  | 0.522 |
| No | 339 (68.2%) | 138 (65.4%) |  |
| Yes | 158 (31.8%) | 73 (34.6%) |  |
| Smoking Hx, n (%) |  |  | 0.593 |
| No | 385 (77.5%) | 168 (79.3%) |  |
| Yes | 112 (22.5%) | 43 (20.4%) |  |
| Sleep Duration (hours) | 8.0 (7.0, 8.0) | 8.0 (7.0, 8.0) | 0.882 |
| Staple Food Freq, n (%) |  |  | 0.551 |
| 1-2 bowls/day | 336 (67.6%) | 135 (64.0%) |  |
| 3-4 bowls/day | 140 (28.2%) | 68 (32.2%) |  |
| 5-6 bowls/day | 21 (4.2%) | 8 (3.8%) |  |
| Vegetable Intake Freq, n (%) |  |  | 0.477 |
| No intake/<1 plate/day | 5 (1.0%) | 4 (1.9%) |  |
| 1 plate/day | 254 (51.1%) | 107 (50.7%) |  |
| 2 plates/day | 154 (31.0%) | 73 (34.6%) |  |
| ≥3 plates/day | 83 (16.7%) | 27 (12.8%) |  |
| ≥4 plates/day | 1 (0.2%) | 0 (0.0%) |  |
| Fruit Intake Freq, n (%) |  |  | 0.211 |
| No intake/<1 serving/day | 12 (2.4%) | 10 (4.7%) |  |
| 1 serving/day | 302 (60.8%) | 115 (54.5%) |  |
| 2 servings/day | 157 (31.6%) | 76 (36.0%) |  |
| ≥3 servings/day | 26 (5.2%) | 10 (4.7%) |  |
| Dairy/Milk Tea Freq, n (%) |  |  | 0.917 |
| No intake/<1 serving/week | 49 (9.9%) | 18 (8.5%) |  |
| 1-3 servings/week | 314 (63.2%) | 133 (63.0%) |  |
| 4-6 servings/week | 68 (13.7%) | 32 (15.2%) |  |
| ≥3 servings/day | 66 (13.3%) | 28 (13.3%) |  |
| Sodium Pilaf, n (%) |  |  | 0.449 |
| Low | 133 (26.8%) | 50 (23.7%) |  |
| High | 364 (73.2%) | 161 (76.3%) |  |
| Sodium Naan, n (%) |  |  | 0.100 |
| Low | 252 (50.7%) | 92 (43.6%) |  |
| High | 245 (49.3%) | 119 (56.4%) |  |
| Sodium Pickled Food, n (%) |  |  | 0.383 |
| Low | 357 (71.8%) | 159 (75.4%) |  |
| High | 140 (28.2%) | 52 (24.6%) |  |
| Household Income (RMB/year), n (%) |  |  | 0.515 |
| < 4000 | 87 (17.5%) | 37 (17.5%) |  |
| 4000-9999 | 39 (7.8%) | 10 (4.7%) |  |
| 10000-20000 | 41 (8.2%) | 19 (9.0%) |  |
| > 20000 | 330 (66.4%) | 145 (68.7%) |  |
| AMC (×10⁹/L) | 0.4 (0.3, 0.4) | 0.4 (0.3, 0.4) | 0.661 |
| P-LCR (%) | 25.3 (20.4, 30.4) | 25.1 (21.4, 30.1) | 0.507 |
| A/G Ratio | 2.1 (1.9, 2.3) | 2.1 (1.9, 2.3) | 0.789 |
| MCH (pg) | 29.6 (28.5, 30.7) | 29.5 (28.3, 30.6) | 0.325 |
| ALB (g/L) | 47.6 ± 2.4 | 47.7 (46.3, 49.4) | 0.916 |
| DBIL (μmol/L) | 2.8 (2.2, 3.8) | 2.9 (2.3, 4.2) | 0.155 |
| TG (mmol/L) | 1.7 (1.1, 2.5) | 1.5 (1.0, 2.2) | 0.032 |

Abbreviations: SBP, Systolic Blood Pressure; DBP, Diastolic Blood Pressure; WC, Waist Circumference; HC, Hip Circumference; PA, Physical Activity; SSB, Sugar-Sweetened Beverage; Hx, History; AMC, Absolute Monocyte Count; P‑LCR, Platelet‑Large Cell Ratio; A/G Ratio, Albumin/Globulin Ratio; MCH, Mean Corpuscular Hemoglobin; ALB, Albumin; DBIL, Direct Bilirubin; TG, Triglycerides.

**Supplementary Table S2. Methodological specifications and XGBoost hyperparameters**

| **Category** | **Item** | **Setting** |
| --- | --- | --- |
| Study design | Data split | 7:3 stratified random split (stratified by outcome) |
|  | Training / Validation size | 497 / 211 |
|  | Final predictors | 9 |
| Preprocessing | Resampling | SMOTE oversampling (training set only) |
|  | Standardization | Z-score (training set mean and SD only) |
|  | Feature selection | LASSO + XGBoost gain-based ranking |
|  | Calibration | Platt scaling (primary); isotonic regression (exploration) |
|  | Evaluation metrics | AUC, Accuracy, Sensitivity, Specificity, Brier score, Calibration slope, HL test, DCA |
|  | Cross-validation | 10-fold |
|  | Random seed | 123 |
| Tuning grid | nrounds | 50, 100, 150 |
|  | max_depth | 2, 3 |
|  | eta | 0.01, 0.03, 0.05 |
|  | gamma | 0 |
|  | colsample_bytree | 0.8 |
|  | min_child_weight | 1 |
|  | subsample | 0.8 |
| Final optimal model | objective | binary:logistic |
|  | eval_metric | auc |
|  | max_depth | 3 |
|  | eta | 0.03 |
|  | nrounds | 100 (early_stopping_rounds = 20) |
| Software | R version | 4.4.3 |
|  | Core packages | xgboost v3.2.1.1, caret v7.0.1, pROC v1.19.0.1 |

All preprocessing, feature selection and hyperparameter tuning were conducted exclusively within training folds to eliminate data leakage; nested cross-validation was not adopted.

**Supplementary Table S3. Key threshold-based predictive performance of the XGBoost model in the validation set**

| **Threshold** | **Accuracy** | **Sensitivity** | **Specificity** | **PPV** | **NPV** | **F1-score** | **Note** |
| --- | --- | --- | --- | --- | --- | --- | --- |
| 0.500 | 0.692 | 0.688 | 0.704 | 0.871 | 0.437 | 0.769 | Reference cutoff |
| 0.538 | 0.682 | 0.650 | 0.778 | 0.895 | 0.433 | 0.753 | Optimal (Youden index) |
| 0.670 | 0.673 | 0.631 | 0.796 | 0.900 | 0.426 | 0.742 | Recommended clinical threshold |

All metrics calculated based on Platt-scaled probabilities. Global calibration metrics: Brier score=0.208, calibration slope=0.720, calibration intercept=1.07. PPV, Positive Predictive Value; NPV, Negative Predictive Value. These thresholds are exploratory and derived solely from internal validation; prospective external validation is required before clinical application.

**Supplementary Table S4. Feature importance and simplified 20-point risk scoring system**

| **Predictive Variable** | **Total Gain** | **Simplified Score** |
| --- | --- | --- |
| Age | 0.345 | 7 |
| Education level | 0.253 | 5 |
| Hip Circumference (HC) | 0.083 | 2 |
| High sodium pilaf intake | 0.073 | 1 |
| Platelet-Large Cell Ratio (P-LCR) | 0.073 | 1 |
| Direct Bilirubin (DBIL) | 0.073 | 1 |
| Systolic Blood Pressure (SBP) | 0.049 | 1 |
| Triglycerides (TG) | 0.040 | 1 |
| Sleep Duration | 0.011 | 0 |

Gain reflects feature predictive contribution. Simplified scores normalized to 0–20 range. Validation in the validation set: AUC of the simplified score = 0.594; Pearson correlation with original XGBoost probability = 0.201; AUC of the full XGBoost model = 0.747. The simplified score is intended as a proposed auxiliary tool for rough screening without computational support. This simplified scoring system has not been prospectively validated and should be considered exploratory pending independent external validation. Abbreviations: HC, Hip Circumference; P‑LCR, Platelet‑Large Cell Ratio; DBIL, Direct Bilirubin; SBP, Systolic Blood Pressure; TG, Triglycerides.

**Supplementary Table S5. Risk stratification scheme and clinical implementation workflow**

| **Risk Level** | **Probability Range** | **Actual Prevalence** | **Recommended Management** |
| --- | --- | --- | --- |
| Low Risk | 0 – 0.357 | 55.7% | Routine health education; annual cognitive follow-up |
| Moderate Risk | 0.357 – 0.770 | 77.4% | Lifestyle intervention; re-screen within 6–12 months |
| High Risk | 0.770 – 1.000 | 92.9% | Urgent referral for comprehensive neuropsychological assessment |

**Risk stratification scheme and standardized implementation steps**

Collect 9 routine variables (5–10 min)

1.Calculate the calibrated XGBoost‑predicted probability (1–2 min)

2.Stratify patients into three risk groups

3.Deliver tiered intervention guidance

4.Establish long‑term electronic follow‑up archives

*Footnote:* Probability cut‑offs derived from calibrated XGBoost predictions in the validation set. All nine indicators can be obtained from routine rural annual physical examination records. These stratification thresholds are exploratory and prospective external validation is required before clinical deployment.

**Supplementary Figures**

**Supplementary Figure S1. CONSORT-style participant flow diagram**

The diagram illustrates the participant screening process from completion of routine health examinations to final analysis inclusion. Of the 711 residents who completed all routine health examination items, all were invited to participate in the MoCA cognitive assessment; 708 (99.6%) agreed and completed the full assessment and were included in the final analysis, while 3 declined to participate.

**Supplementary Figure S2. Decision curve analysis for the XGBoost model in the validation set**

The y-axis represents net benefit, and the x-axis represents the high-risk threshold probability. The model curve shows the net benefit of using XGBoost for screening compared with the "screen all" and "screen none" strategies. The XGBoost model provided positive net benefit across threshold probabilities of approximately 0.1 to 0.8, supporting its clinical utility for primary care screening.

**Supplementary Figure S3. Clinical workflow diagram for primary care implementation**

Primary care physicians collect the nine readily available variables (Step 1), calculate the calibrated XGBoost predicted probability (Step 2), stratify patients into low‑risk (<0.357), moderate‑risk (0.357–0.770), and high‑risk (>0.770) categories (Step 3), deliver tiered interventions accordingly (Step 4), and establish long‑term follow‑up records for cognitive monitoring (Step 5). Abbreviations: SBP, systolic blood pressure; HC, hip circumference; DBIL, direct bilirubin; TG, triglycerides; P‑LCR, platelet‑large cell ratio.
